# Experimental guide wire placement for total shoulder arthroplasty in glenoid models: higher precision for patient-specific aiming guides compared to standard technique without learning curve

**DOI:** 10.1186/s12891-024-07549-0

**Published:** 2024-06-06

**Authors:** Jana F. Schader, Tobias Helfen, Volker Braunstein, Ben Ockert, Florian Haasters, Ralph Hertel, Norbert Südkamp, Stefan Milz, Christoph M. Sprecher

**Affiliations:** 1 AO Research Institute Davos, Clavadelerstrasse 8, Davos Platz, 7270 Switzerland; 2 Department of Orthopedics, Balgrist University Hospital, University of Zurich, Forchstrasse 340, Zurich, 8008 Switzerland; 3 Department of Orthopaedics and Trauma Surgery, Musculoskeletal University Center Munich (MUM), LMU Munich, Marchioninistr. 15, 81377 Munich, Germany; 4 OrthoPlus München, Alte Börse, Lenbachplatz 2a, 80333 Munich, Germany; 5 Schön Klinik München Harlaching, Zentrum für Knie-, Hüft- und Schulterchirurgie, Harlachinger Strasse 51, 81547 Munich, Germany; 6 Schulter & Ellbogen Zentrum Bern, Lindenhofspital, Bremgartenstrasse 117, Bern, 3001 Switzerland; 7 Medical Faculty, Albert-Ludwigs-University Freiburg, 79085 Fahnenbergplatz, Freiburg im Breisgau Germany; 8 Anatomische Anstalt der Ludwig-Maximilians-Universität, Pettenkoferstrasse 11, 80336 Munich, Germany

**Keywords:** Shoulder, Arthroplasty, Glenoid deformity, Patient-specific, Guide, Learning curve, Pre-operative planning

## Abstract

**Background:**

Patient-specific aiming devices (PSAD) may improve precision and accuracy of glenoid component positioning in total shoulder arthroplasty, especially in degenerative glenoids. The aim of this study was to compare precision and accuracy of guide wire positioning into different glenoid models using a PSAD versus a standard guide.

**Methods:**

Three experienced shoulder surgeons inserted 2.5 mm K-wires into polyurethane cast glenoid models of type Walch A, B and C (in total 180 models). Every surgeon placed guide wires into 10 glenoids of each type with a standard guide by DePuy Synthes in group (I) and with a PSAD in group (II). Deviation from planned version, inclination and entry point was measured, as well as investigation of a possible learning curve.

**Results:**

Maximal deviation in version in B- and C-glenoids in (I) was 20.3° versus 4.8° in (II) (*p* < 0.001) and in inclination was 20.0° in (I) versus 3.7° in (II) (*p* < 0.001). For B-glenoid, more than 50% of the guide wires in (I) had a version deviation between 11.9° and 20.3° compared to ≤ 2.2° in (II) (*p* < 0.001). 50% of B- and C-glenoids in (I) showed a median inclination deviation of 4.6° (0.0°-20.0°; *p* < 0.001) versus 1.8° (0.0°-4.0°; *p* < 0.001) in (II). Deviation from the entry point was always less than 5.0 mm when using PSAD compared to a maximum of 7.7 mm with the standard guide and was most pronounced in type C (*p* < 0.001).

**Conclusion:**

PSAD enhance precision and accuracy of guide wire placement particularly for deformed B and C type glenoids compared to a standard guide in vitro. There was no learning curve for PSAD. However, findings of this study cannot be directly translated to the clinical reality and require further corroboration.

## Introduction

Total shoulder arthroplasty (TSA) and reverse total shoulder arthroplasty (rTSA) have been valid treatment options for shoulder arthritis. Accurate glenoid component placement is crucial in shoulder arthroplasty [1], as incorrect implantation poses risk of reduced mobility, inferior notching, increased component wear, implant loosening or dislocation, which may ultimately result in revision surgery [2–5]. Ho et al. found osteolysis around the centre peg of a glenoid component correlating with component retroversion of ≥ 15° [6]. Moreover, it is challenging for surgeons to place a pegged glenoid component perpendicular to the plane of the scapula by asymmetric reaming without centre peg perforation in glenoids exceeding retroversion of 20° [7]. Superior tilt in concentric lateralized glenosphere designs may lead to increased shear forces resulting in component loosening [8]. Nowadays, prosthesis designs allow the surgeon to adapt version and inclination of the glenoid component in order to correct degenerative or traumatic anatomical variations of the glenoid [9].

Walch et al. [10] distinguished three main types of glenoid anatomy in primary shoulder osteoarthritis: type A, B and C. A centralized humeral head produces an equal balance of forces acting on the glenoid in glenoid type A (59%). In glenoid type B (32%) an asymmetrical posterior force distribution on the glenoid leads to a posterior subluxation of the humeral head. Glenoids exhibiting a retroversion greater than 25 degrees are defined as Type C (9%) [10]. Over the years this classification has been modified and specified in more detail [11, 12]. Therefore, the glenoid type plays a major role for pre-operative planning.

A crucial surgical step remains in the positioning of the central glenoidal guide wire for correct placement of the anatomic glenoid component or baseplate component. Variation of guide wire positioning depends on estimation and experience of the surgeon and shows significant variation [13]. Particularly differentiating between precision (variance in hitting a target repeatedly) and accuracy (deviation from the target) has become relevant in evaluating the outcome of surgical navigation devices and might play a crucial regarding the learning curve of surgeons with a lower volume of shoulder arthroplasty [14].

Over the past decade, patient-specific guides and aiming devices evolved in order to improve correct positioning of the central glenoidal guide wire. Various targeting guide designs have been developed ranging from 3D-printed patient-specific single-use guides to reusable generic instrumentation guides to assist the surgeon in positioning the guide wire [15]. However, improvement of guide wire placement with regards to variation of angle and position of the K-wire by the use of a patient-specific aiming device in different glenoid types has been investigated only to a limited extent to date.

The aim of this study was to compare the precision and accuracy of glenoid guide wire positioning in different glenoid prototype models using a standard guide or a patient-specific aiming device, hypothesizing a superior outcome of the patient-specific aiming device over the standard guide. Furthermore, a possible learning curve for the patient-specific aiming device was investigated.

## Materials and methods

**Fig. 1:**
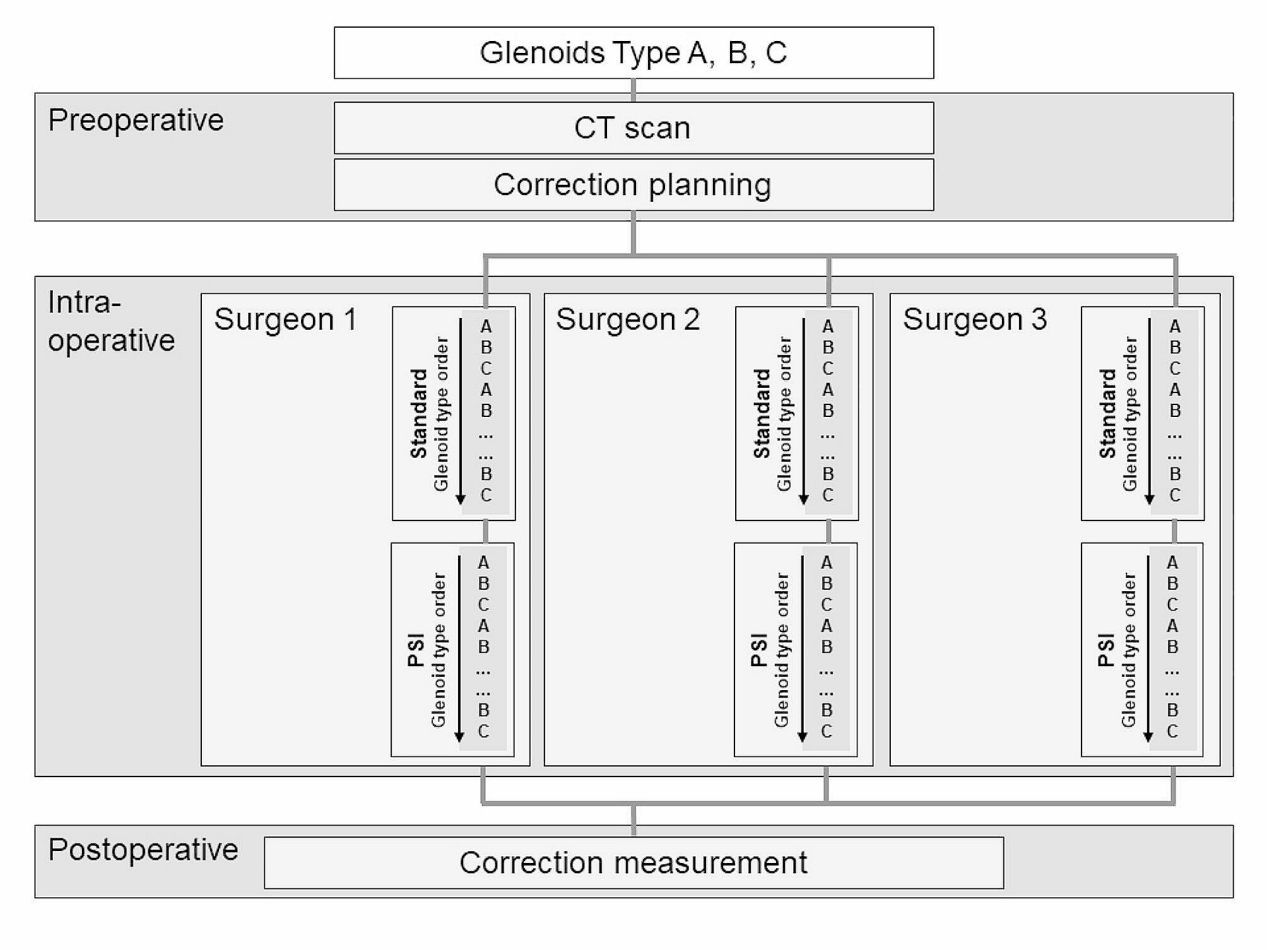
Workflow of guide wire placement in predefined corrections angles for version and inclination into 30 glenoid models of each glenoid type by three experiences surgeons

An experimental study was conducted to assess the precision of guide wire placement into three different glenoid models with a standard guide in group (I) compared to a patient-specific aiming device in group (II). Three experienced surgeons (> 50 shoulder arthroplasties per year) [13] inserted 2.5 mm K-wires, which is the most common guide wire size for pegged glenoid component, in predefined corrections angles for version and inclination into 30 glenoid models of each glenoid type. Therefore, 90 guide wires were placed in each group (Fig. 1).

**Fig. 2:**
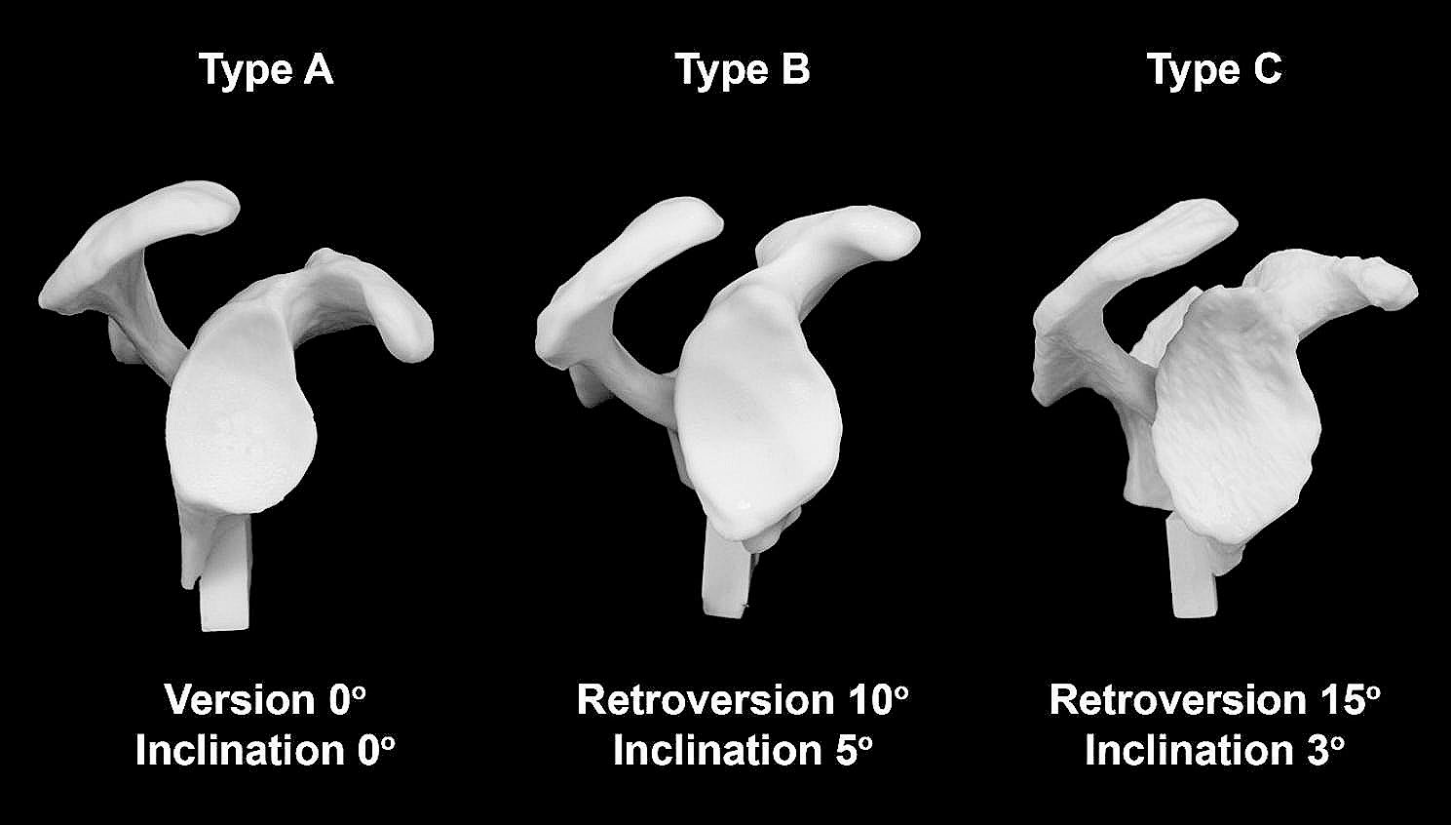
Visualization of different glenoid cast models type A, B and C according to the classification of Walch et al. [10] based on clinical computed tomography (CT) data with planned correction angles for retroversion and inclination

These polyurethane cast glenoid models were vacuum casted from negatives of 3D-printed computed tomographic (CT) scans of human glenoids (Müri Prototech, Gontenschwil, Switzerland). Correction angles were defined in a standard manner in the “pre-operative” planning based on these CT scans. According to the classification of different glenoid morphologies in primary shoulder osteoarthritis by Walch et al. [10], there were 10 Walch type A (predefined correction angles: inclination 0°, version 0°), 10 Walch type B (predefined correction angles: inclination 5°, retroversion 10°) and 10 Walch type C (predefined correction angles: inclination 3°, retroversion 15°) models (Fig. 2). Glenoid models were covered in a standardized fashion with an artificial tissue in order to simulate the anatomic limited intraoperative field without landmarks.

**Fig. 3:**
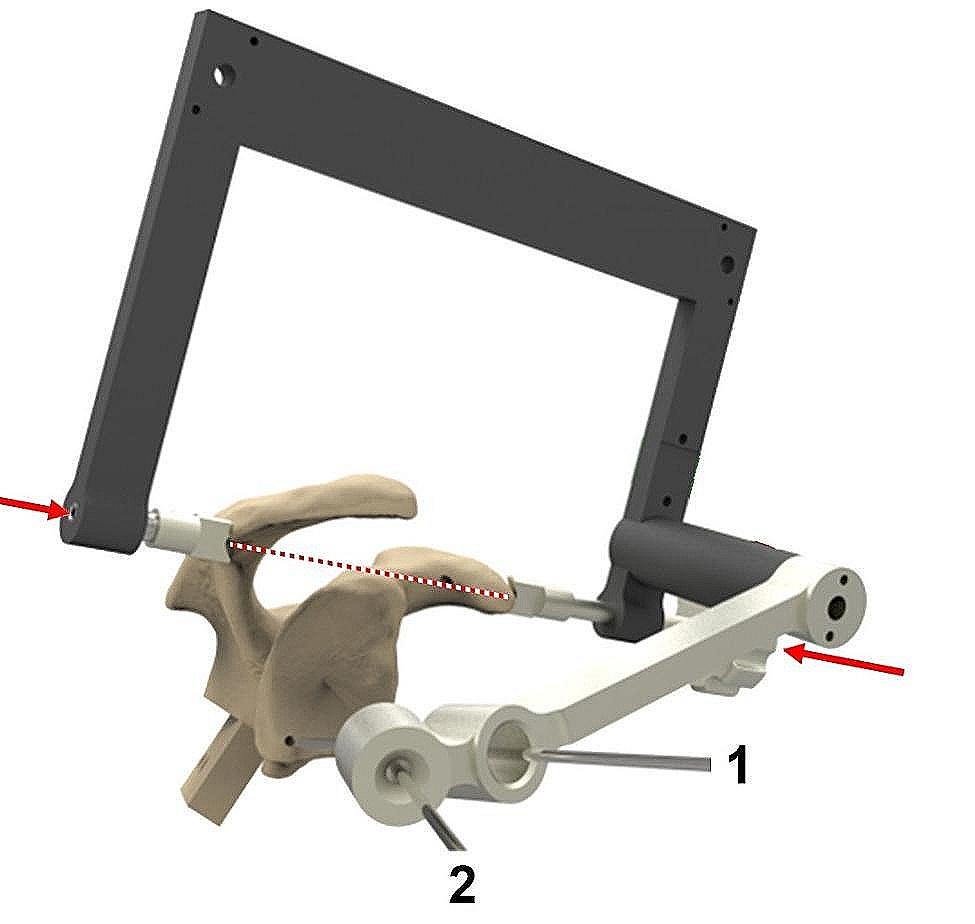
Schematic illustration of patient-specific aiming device (PSAD) mounted via the universal (black) handle on the tip of the coracoid and posterolateral acromion. The control K-wire (1) is inserted through the sleeve of the patient-specific (white) handle onto the inferior point of glenoid fossa of polyurethane cast glenoid model type B. An exemplified guide wire (2) points to the glenoid centre in predetermined corrected angles based on previous 3D CT planning. Soft tissue cover is not displayed

In group (I) a standard guide (Epoca Drill Guide, DePuy Synthes, Oberdorf, Switzerland) was used according to the manufacturer’s guidelines. For group (II) a patient-specific aiming device was constructed in-house, with two components (Fig. 3). One patient-specific handle to place the guide wire through a sleeve perpendicular to the glenoid surface within the aforementioned specific correction angles into the different glenoid types. And one universal handle for the surgeon to position the whole guide on the “fulcrum axis” (a virtual line between the tip of the coracoid and posterolateral acromion). This axis has been described in previous studies as useful landmark for intraoperative evaluation of glenoid version while performing total shoulder arthroplasties [16]. As a third fix point the sleeve of the control K-wire was fixed to the inferior point of the glenoid fossa. The clinical applicability of the patient-specific aiming device was tested in a cadaver lab beforehand. However, the authors want to stress that this device is not intended for commercial use.

The instrumentation was performed by every surgeon with the standard guide in glenoid type A, B and C. This order was repeated ten times by each of them. Subsequently, the exact procedure was performed by every surgeon with the patient-specific aiming device for each glenoid type, respectively (Fig. 1). Each surgeon had one attempt for each guide wire only. The course of all ten trials in each glenoid model performed by each of the three surgeons was analysed for possible learning curves by the split-group method [17]. Data points were split into two data sets with equal sizes (the first and the second half) for both methods (standard and patient specific) and all three glenoid types (A, B, C).

Accuracy was defined as deviation from the planned entry point, whereas precision was defined as variance in version and inclination of the guide wire in glenoids type A, B and C and has been measured by a visual measurement system (Measuring Projector, Type PJ 300, Mitutoyo, Kawasaki, Kanagawa, Japan).

### Statistical analysis

Statistical analysis was performed with SPSS software package (IBM SPSS Statistics, V23, IBM, Armonk, NY). Shapiro-Wilk test was conducted to screen the data for normality of distribution. Significant differences between the two groups regarding version, inclination, distance of the entry point and data of the split-group method for investigating possible learning curves were identified with Paired-Samples t-tests and Related-Samples Wilcoxon Signed Rank Test for non-normally distributed values. Level of significance was set to 0.05 for all statistical tests.

## Results

**Fig. 4:**
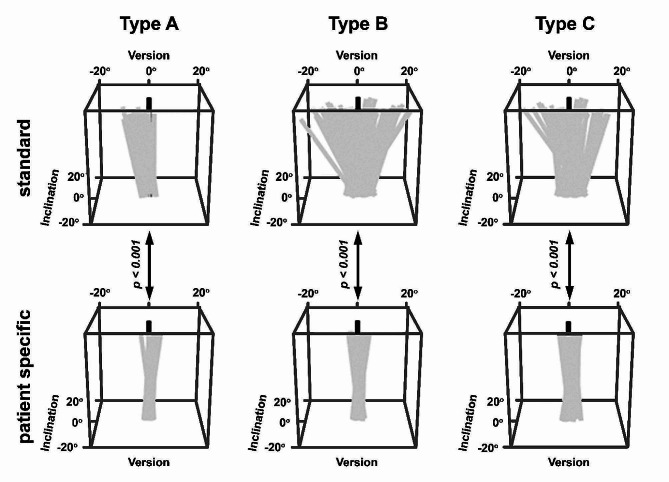
Comparison of guide wire (grey) deviation in version and inclination in degrees from planned position (black) in glenoid types A, B, C (Walch et al. [10]). Deviation and range are significantly larger (*p* < 0.001) using the standard guide (first row) compared to the use of a patient-specific aiming device (second row) independent of the glenoid type. However, largest difference is seen in glenoid types B and C. Base plate of cuboid symbolizing glenoid surface

Deviation of all inserted guide wires of both groups from the planned position is illustrated in Fig. 4 as 3D graphics and described in Table 1.

**Table 1:** Illustration of deviation in version and inclination of all inserted guide wires of both groups from the planned position in glenoid types A-C and overall, independent of glenoid type

	Type A		Type B		Type C		Types A, B, C	
	standard	Patient specific	standard	Patient specific	standard	Patient specific	standard	Patient specific
Version	3.2 (0.2–10.1)	1.0 (0.0–4.0)	11.9 (0.1–20.3)	2.2 (0.0–4.8)	7.4 (0.9–17.3)	1.3 (0.0–3.8)	6.9 (0.1–20.3)	1.4 (0.0–4.8)
	*p* < 0.001		*p* < 0.001		*p* < 0.001		*p* < 0.001	
Inclination	5.7 (0.0–14.6)	1.2 (0.0–4.4)	5.3 (0.4–10.1)	1.9 (0.0–4.0)	4.3 (0.0–20.0)	1.8 (0.0–3.7)	4.8 (0.0–20.0)	1.7 (0.0–4.4)
	*p* < 0.001		*p* < 0.001		*p* < 0.001		*p* < 0.001	

## Glenoid version

**Fig. 5:**
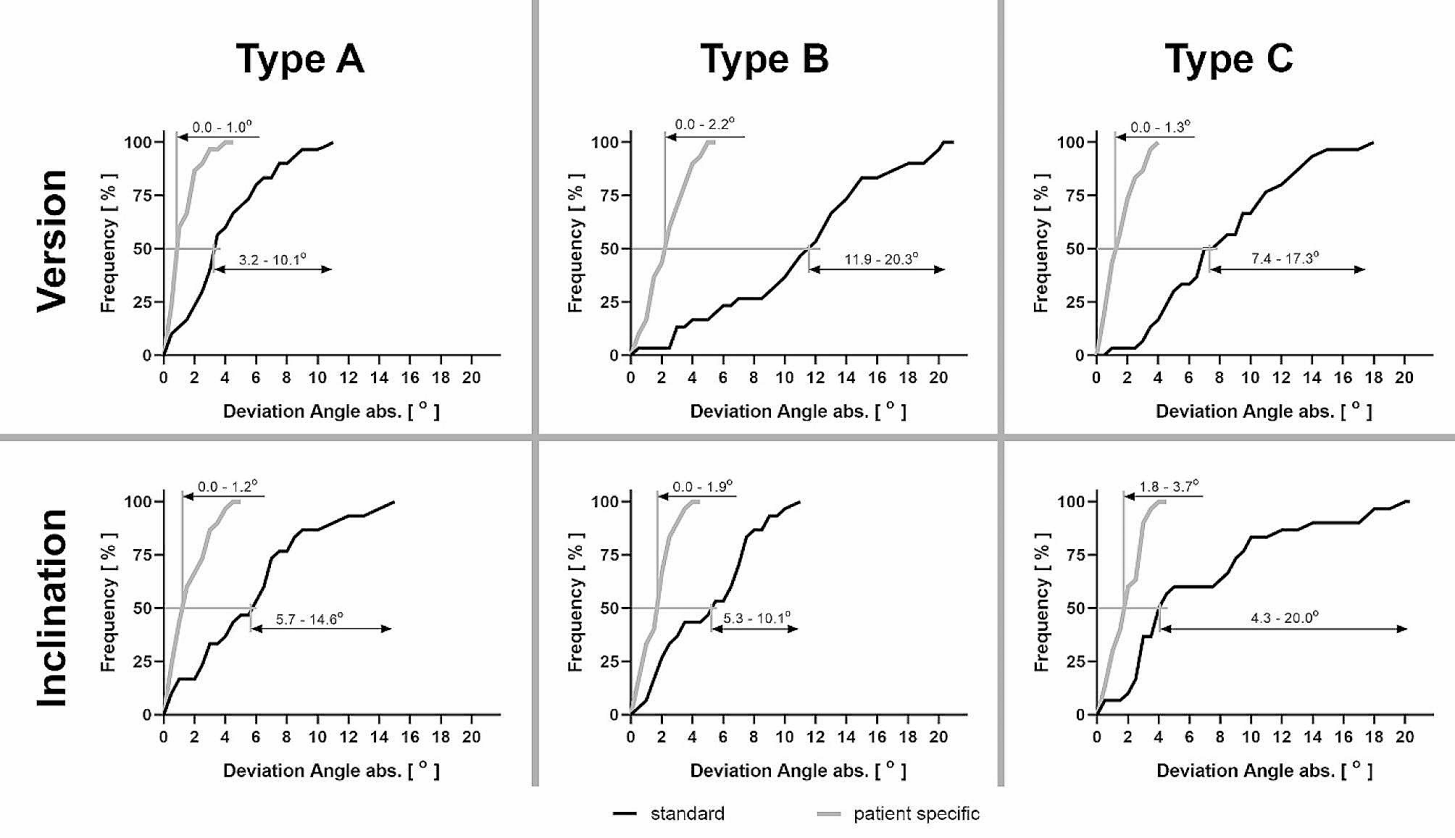
Cumulated histogram presenting absolute deviation angle, regarding version and inclination, from planned insertion into glenoid types A, B and C by standard guide (black line) or patient-specific aiming device (grey line). Median version and inclination angle at 50% frequency threshold displayed with range

The maximal deviation from the planned glenoid version correction when using the patient-specific aiming device was 4.0°, 4.8° and 3.8° for glenoid type A, B and C, respectively, compared to 10.1°, 20.3° and 17.3° in (I) (*p* < 0.001) (Fig. 5). Deviation of version in greater than 50% of A-glenoids (I) ranged between 3.2° and 10.1° in (I) compared to ≤ 1.0° in (II) (*p* < 0.001). For glenoid type B, more than 50% of the guide wires placed in (I) had a median deviation of ≥ 11.9° (range: 0.1° – 20.3°; *p* < 0.001) compared to ≤ 2.2° (range: 0.0° – 4.8°; *p* < 0.001) with the patient-specific aiming device (Fig. 5). Deviation of version in greater than 50% of C-glenoids (I) ranged between 7.4° and 17.3° compared to ≤ 1.3° in (II) (*p* < 0.001). Overall, deviation of version using the patient-specific aiming device was smaller in all three types of glenoids with 1.4° (range: 0.0° – 4.8°; *p* < 0.001) compared to 6.9° (range: 0.1° – 20.3°; *p* < 0.001).

### Glenoid inclination

The maximal deviation from the planned glenoid inclination correction when using the patient-specific aiming device was 4.4°, 4.0° and 3.7° for glenoid type A, B and C, respectively, compared to 14.6°, 10.1° and 20.0° in (I) (*p* < 0.001) (Fig. 5). In > 50% of A-glenoids in (I) deviation was between 5.7° and 14.6° compared to ≤ 1.2° in (II) (*p* < 0.001). In glenoid type B, deviation of inclination of more than 50% of the guide wires placed in (I) ranged between 5.3° and 10.1° compared to ≤ 1.9° in (II); in C-glenoids between 4.3° and 20.0° in (I) compared to ≤ 1.8° in (II) (*p* < 0.001). Overall, deviation of inclination using the patient-specific aiming device was smaller in all three types of glenoids with 1.7° (range: 0.0° – 4.4°; *p* < 0.001) compared to 4.8° (range: 0.0° – 20.0°; *p* < 0.001).

### Glenoid entry point

**Fig. 6:**
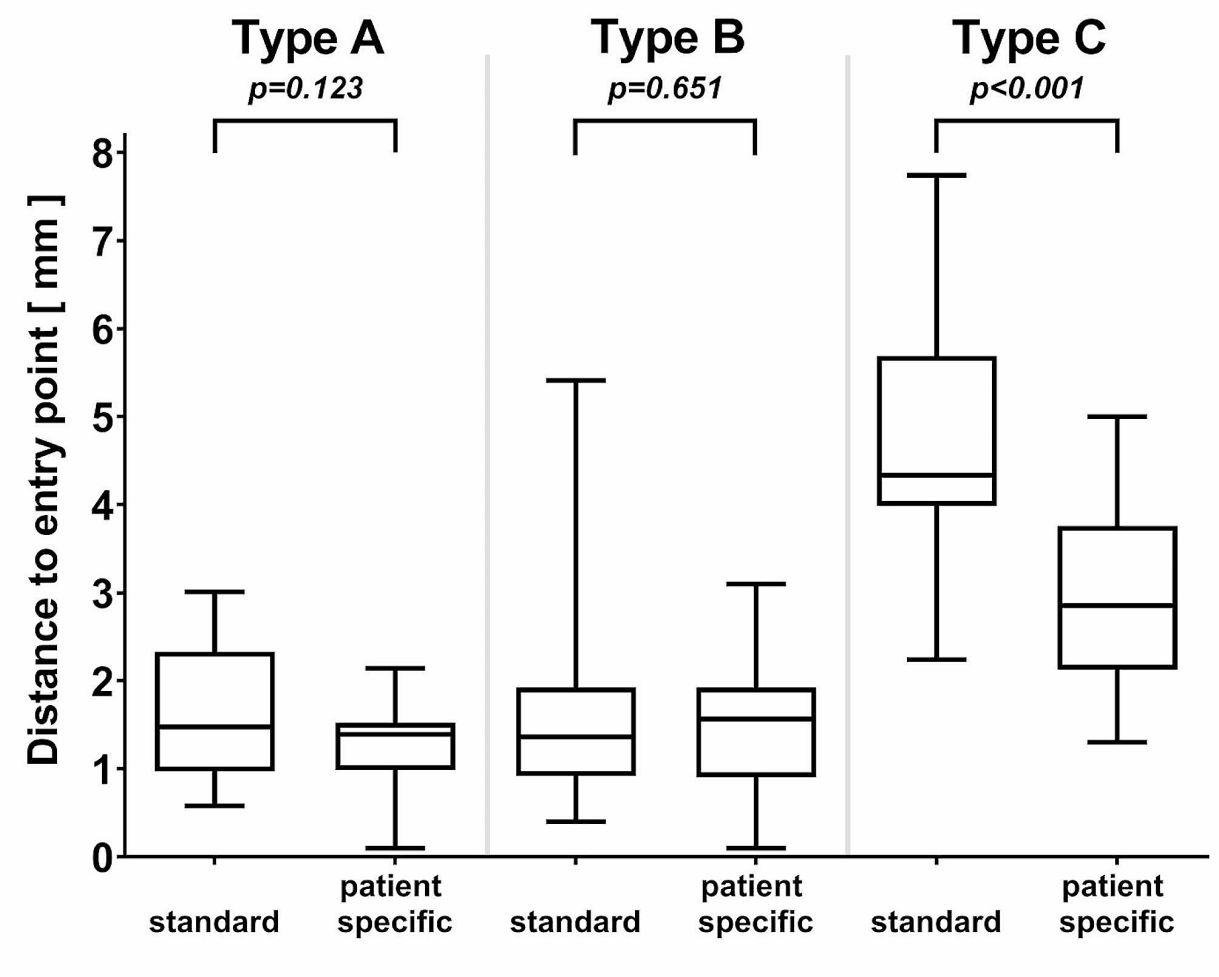
Diagram presenting absolute distance from planned entry point of the guide wires in different glenoid models attained with standard guide and patient-specific aiming device. Difference in accuracy between patient-specific aiming device and standard guide was most pronounced for type C (*p* < 0.001)

In group I, the probability of hitting the planned entry point decreased from glenoid types A to C (Fig. 6). A larger scattering of the entry point was observed in group (I) in each glenoid, with a maximum in type C (Fig. 6). In more detail, deviation from the entry point was in all glenoid types less than 5.0 mm when using the patient-specific aiming device compared to a maximum of 7.7 mm with the standard guide. Difference in accuracy between patient-specific aiming device and standard guide was most pronounced for type C (*p* < 0.001).

### Learning curve

**Fig. 7:**
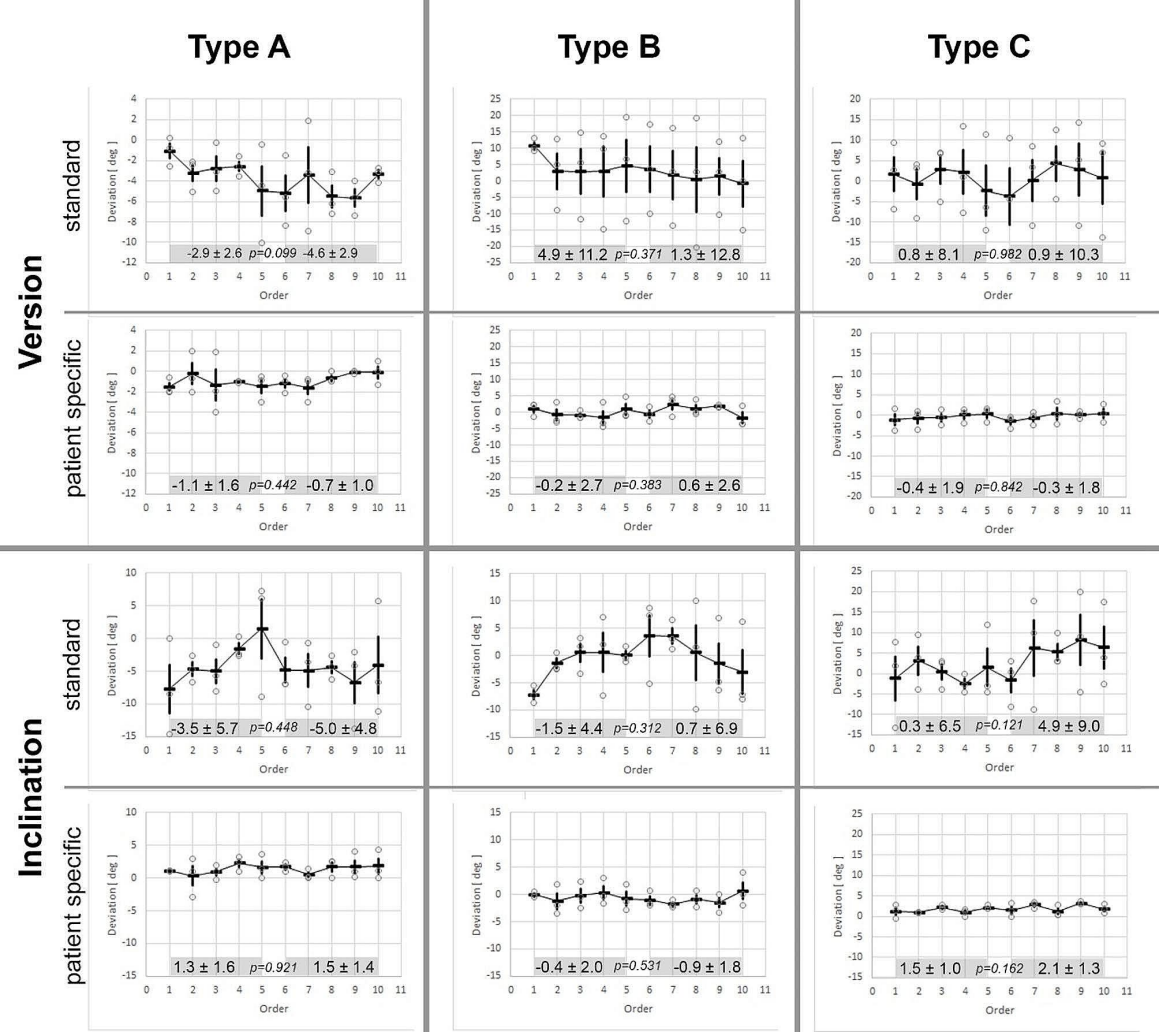
Deviation of version and inclination achieved by standard guide compared to patient-specific aiming device (PSAD) of all three surgeons over the course of all ten trials in each glenoid model. Smaller variance in deviation of version and inclination achieved by PSAD compared to standard guide. Using the split-group method for analysis, there was no learning curve with neither of the devices (values depicted as mean ± standard deviation, *p-value*)

Comparing the first half of trials with the second, according to the split-group method, no significant change of deviation of version and inclination of the k-wire placed with the patient-specific aiming device by each of three experienced surgeons was shown over the course of ten trials for each glenoid type (Fig. 7). This observation was consistent with the standard guide; however, a greater variance in the deviation of version and inclination of the K-wire was noted among individual surgeons.

## Discussion

In this study, 180 guide wires were inserted into three different types of polyurethane cast glenoid models acquired from arthritic glenoid CT scans by three surgeons in a standardized experimental set-up. The precision of the guide wire placement regarding version, inclination and entry point was significantly higher with the patient-specific aiming device compared to the standard guide in all three types of glenoids, especially in worn-out glenoids as type B and C.

These results corroborate with other in-vitro studies observing a significant average deviation in version from the pre-operative plan of ≤ 5° using a patient-specific aiming device compared to ≥ 5° with standard guide [18–22]. Also various in-vivo studies showed an improvement of glenoid positioning with PSI [23, 24]. Based on 3D CT scans, correction angles for version and inclination as well as the entry point were defined pre-operatively by one of three surgeons in this study. Accordingly, Iannotti et al. [15] compared the accuracy of glenoid implant placement in primary TSA among different types of guides used with 3D CT pre-operative planning. They observed a greater accuracy in using reusable patient-specific aiming devices compared to standard guides regarding lower frequency of outliers for version > 10°, a higher frequency of outliers for inclination > 5° with standard guides in type A glenoids and less accuracy within all instrumentation groups in glenoids with pre-operative posterior glenoid bone loss or severe glenoid retroversion. However, there was a predominance of nonsignificant differences across most comparisons with regard to the different instrumentation technologies. Another recent pooled analysis [25] showed no significant difference in the accuracy of glenoid component positioning with patient-specific or standard guides. There might be various reasons for this discrepancy of results, the following have been addressed in this study: experience of the surgeon, intraoperative exposure and glenoid morphology. However, the authors like to stress that the study did not investigate the applicability of this specific patient-specific aiming device, but rather precision and accuracy of a patient-specific aiming device concept compared to standard devices in general.

Three experienced shoulder surgeons, performing > 50 shoulder arthroplasties/ year [13], inserted 30 guide wires each in a standardized fashion into glenoid cast models. They have never worked with the used patient-specific aiming device before and therefore had no experience in the application of the guide. Nevertheless, all of the surgeons achieved a higher precision as well as accuracy in positioning the guide wire compared to the standard guide. Therefore, the assistance of a patient-specific guide might improve precision of glenoid component of every surgeon, especially those with a lower volume of shoulder arthroplasty implantations.

In the clinical reality, glenoid exposure can be challenging, especially in glenoid deformities [26]. There is a variety of different designs of patient-specific guides, a majority is placed directly on the glenoid itself. However, intraoperative exposure might be restricted and placing the guide might lead to soft tissue damage. Also, osteophytes might be resected differently to the pre-operative plan, which could lead to difficulties in translating the pre-operative plan by placing the guide directly onto the glenoid. In this study, a guide was constructed which could be placed on the fulcrum axis, thus no more than the actual exposure for the glenoid component itself was necessary. As mentioned previously, the patient-specific guide should be applicable for all levels of surgeons, especially if the volume for the use of this technology is low. However, the purpose of the study was not the implementation of this specific guide, rather than proving the principle concept of patient-specific guide application in deformed glenoids.

Maximal deviation from the planned glenoid version correction when using the standard guide was 20.3° and more than 50% of the guide wires placed in glenoid type B with the standard guide had a deviation of ≥ 11.9° compared to ≤ 2.2° with the patient-specific aiming device. Regarding inclination, more than 50% of the guide wires placed in group (I) had a maximal deviation of 20.0° for glenoid type B and C. This agrees with other studies showing that in comparison with standard pre-operative planning and instrumentation, patient-specific planning reduces variability in inclination of the glenoid component, as well as the risk of extreme inclination errors for TSA and rTSA [27]. A meta-analysis investigating clinical studies reports a version error of 4.5° (95% CI 2.2° − 6.9°) with PSI compared to 8.6° (95% CI 6.6° − 10.7°), an inclination error of 2.7° (95% CI 1.4 °- 4.1°) compared to 11.1° (95% CI 6.9° − 15.2°) and offset error of 1.9 mm (95% CI 1.0 –2.8 mm) compared to 3.4 mm (95% CI 2.8 –4.0 mm), respectively [28]. Regarding the entry point, there was no significant improvement of guide wire placement with the patient-specific device in glenoids A and B and a larger deviation from the planned entry point in glenoid type C. However, there was a significant difference of the entry point achieved by the standard guide and the patient-specific guide in glenoid type C. These results are interpreted in the context of the extensively worn surface of the type C glenoid model, which may lead to a more difficult placement of the guide wire on the surface which can be improved by using a patient-specific device. These findings corroborate with other studies describing more accurate glenoid implant placement with patient-specific instrumentation in more severe bone deformity [1, 18]. One study also investigated reliability and precision of PSI compared to non-PSI methods in different 3D- printed glenoids models and reported similar results with an overall deviation of the version angle from the plan of 2.68 ± 2.10°, an inclination angle deviation from the plan of 2.59 ± 2.68°, and the deviation of the entry point offset from the plan of 1.55 ± 1.26 mm [29].

Sanz-Ruiz et al. stated that patient specific instrumentation applied in unicompartmental knee arthroplasty may improve precision of component alignment during the learning curve of unexperienced surgeons, thus achieving functional results similar to those of more experienced surgeons using a conventional procedure [30]. Learning curve with PSI in total shoulder arthroplasty in different glenoid types in a standardized in-vitro setting has not been investigated so far. Our study showed no learning curve within each experienced surgeon but higher precision, meaning lower variance of deviation during the course of ten trials for each glenoid model compared to the standard guide. Therefore, PSAD may not only improve accuracy of k-wire placement compared to standard guides, but also increase precision with repeated lower variance of deviation even in experienced surgeons.

### Strengths of the study

As previously mentioned, this study addresses points, which differ from those explored in prior research in the field: experience of the surgeon, intraoperative exposure and glenoid morphology. Our study showed no learning curve within each experienced surgeon but higher precision during the course of ten trials for each glenoid model compared to the standard guide. Furthermore, a guide was constructed which could be placed on the fulcrum axis, thus no more than the actual exposure for the glenoid component itself was necessary. As mentioned previously, the patient-specific guide should be applicable for all levels of surgeons, especially if the volume for the use of this technology is low. Moreover, the study proved that there was a more accurate glenoid implant placement with patient-specific instrumentation in more severe bone deformity even in high volume (experienced) surgeons.

### Limitations of the study

However, interpretation of the findings requires the consideration that this study was performed in vitro to simulate guide wire placement in different types of glenoids. Therefore, the specific findings of this study cannot be directly translated to the clinical reality and require further corroboration. Furthermore, partly inherent to the experimental fashion of the study, we did not assess other important outcomes that could represent learning such as operative time/installation time or complications. Although the split-group method being one of the most common approaches for learning curve assessment with statistical testing, there might be some bias by arbitrarily splitting the group size in two halves [31].

Of course, virtually constructed 3D – printed instruments and implants are cost- and pre-operatively time consuming and their effectiveness has not entirely been proven yet [25]. However, processes of this rapidly emerging technology, which already has been adopted by other specialties like craniomaxillofacial surgery on a frequent basis, are expected to be optimized in the future. Our study showed that their application may improve patient outcome by improving glenoid implant placement especially in selected cases of severe glenoid deformity or even fracture sequelae, which primarily have been treated conservatively [32].

## Conclusion

In vitro, patient-specific aiming devices substantially enhanced the accuracy of guide wire placement and reduced the variation compared to a standard guide. Furthermore, there was no learning curve for PSAD, displaying its high precision.

Particularly for B and C type glenoids, where especially deviation of version in deformed B type glenoids was decreased by a median of 10 degrees with the patient-specific aiming device, the results may translate to the clinical setting, but need to be tested in a clinical patient population.

## Data Availability

The datasets used and/or analysed during the current study are available from the corresponding author on reasonable request.
